# Accuracy assessment of a novel semiautomatic method evaluating bone grafts around the dental implant: an in vitro and ex vivo study

**DOI:** 10.1038/s41598-020-71651-1

**Published:** 2020-09-10

**Authors:** Jun-Yu Shi, Yuan Li, Long-Fei Zhuang, Xiao Zhang, Ling-Feng Fan, Hong-Chang Lai

**Affiliations:** 1 Department of Oral and Maxillofacial Implantology, Shanghai Ninth People’s Hospital, Shanghai Jiao Tong University School of Medicine, 639 Zhizaoju Road, Shanghai, China; 2 National Clinical Research Center for Oral Diseases, Shanghai, China; 3 Shanghai Key Laboratory of Stomatology & Shanghai Research Institute of Stomatology, Shanghai, China; 4 Huishang Dental Clinic, Shanghai, China; 5 Department of Radiology, Shanghai Ninth People’s Hospital, School of Medicine, Shanghai Jiao Tong University, Shanghai, China

**Keywords:** Bone, Software

## Abstract

The present study aimed to evaluate the accuracy and repeatability of morphological contour interpolation (MCI)-based semiautomatic segmentation method for volumetric measurements of bone grafts around dental implants. Three in vitro (one with a cylinder and two with a geometrically complex form) and four ex vivo models (peri-implant cylinder-shaped bone defect) were created for imitating implant placement with simultaneous guided bone regeneration (GBR) procedure. Cone beam computerized tomography (CBCT) scans of all models were obtained with the same parameters. For volumetric measurements, the actual volumes of bone grafts in models were assessed by computer-aided calculation and both manual and MCI-based methods were utilized as test methods. The accuracy of the methods was evaluated by comparing the measured value and the actual volume. The repeatability was assessed by calculating the coefficients of variation of repeated measurements. For the accuracy of three dimensional (3D) reconstructions, the computer-designed corresponding models were set as the reference and the morphological deviation of 3D surface renderings created by two methods were evaluated by comparing with reference. Besides, measurement time was recorded and a comparison between the two methods was performed. High accuracy of the MCI-based segmentation method was found with a discrepancy between the measured value and actual value never exceeding − 7.5%. The excellent repeatability was shown with coefficients of variation never exceeding 1.2%. The MCI-based method showed less measurement time than the manual method and its 3D surface rendering showed a lower deviation from the reference.

## Introduction

The clinical success of implant treatment is strongly associated with adequate bone volume, which allows the position of the implant in a biologically accepted and prosthetically driven location^1^. Mispositioned implants due to insufficient bone volume can lead to esthetic failure. It is reported that 60% of implants need additional bone augmentation procedures to achieve good esthetics^2^. Guided bone regeneration (GBR) is the most widely used and well-documented procedure to augment bone in localized alveolar defects^3–5^.

So far histological evaluation is the most accurate method to characterize the fate of grafted bone over time^6^. However, it cannot be applied in clinical routine and non-invasive methods have been proposed for this purpose^7,8^. Previous studies have described methods for volumetric assessment of augmented regions included optical scanning^9–12^ and cone beam computerized tomography (CBCT) image-based measurement^13,14^. The main limitations of optical scanning are that they only provide information about the lateral contour precluding the analyses of the interior contour, such as the morphology of initial bone defect. And it must be taken into consideration that the accuracy of digital three dimensional (3D) matching procedures is not sufficiently investigated^8^.

CBCT is currently considered a well-established adjunctive diagnostic, virtual simulation, and treatment planning tool with various clinical applications in implant dentistry^15^. Linear measurements of the thickness of grafted facial bone wall around dental implants in cross-sectional images of CBCT are performed in most clinical studies. It is recommended, however, that the measurements should not be limited to the assessment based on the 3D volume in the entire region of interest^8^.

For isolation of region of interest in CBCT imaging, conventional manual segmentation method has been widely used to perform 3D measurements of grafted bone volume^13,14,16^. Although manually created segmentation is currently considered as the gold standard, this segmentation process can be very time-consuming and arduous which may not be clinically applicable. Moreover, the presence of metal artifacts in CBCT images, which are caused by titanium implants or metal-ceramic crowns, may influence the accuracy of the segmentation process.

To overcome the limitations regarding accuracy and efficiency, a novel morphology-based approach for the inter-slice interpolation of CBCT or CT datasets has been introduced to reduce the need for repetitive tracing. It has been proven to be highly reliable and accurate to reconstruct synthetic contours as well as anatomical structures^17^. In our previous study^18^, 3D surface reconstruction derived from CBCT with manual isolation and MCI algorithm has been used for volumetric evaluation of grafted bone volume changes in patients receiving implant placement and simultaneous GBR in the esthetic zone. However, the accuracy of this method warrants further research.

Therefore, the aims of this study were (1) to evaluate the accuracy and repeatability of the MCI-based semiautomatic method for volumetric measurements of models imitating implant placement with simultaneous GBR (2) to examine the accuracy of created 3D reconstruction models and the execution time.

## Results

For the cylindrical model (R) and geometrically complex models (S1/S2), the volume representing the control values were computer calculated and listed in Table 1. The errors between the computer calculation and the result of the fabrication were also presented in Table 1.

**Table 1:** The designed volume of bone grafts filled in vitro models and maximum differences between the true volume and the calculated volume, expressed in volume percent, resulting from the imprecision of the fabrication process.

	R	S1	S2
Volume	928.44	954.77	404.82
Error of fabrication (%)	± 0.24	± 0.31	± 0.4

### Repeatability

**Figure 1:**
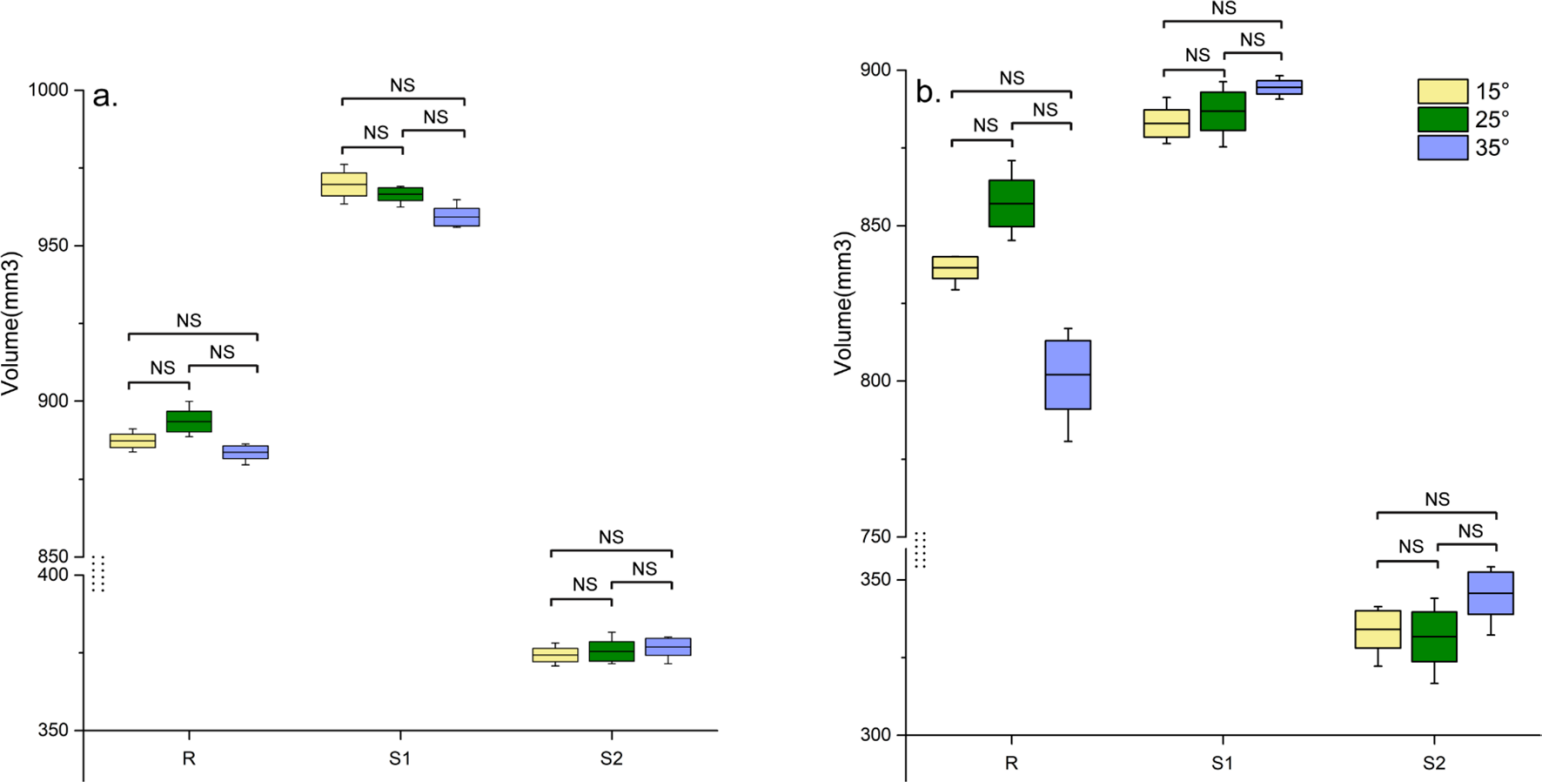
Boxplot of volume measurements of bone grafts filled in models S1–2 and R mounted on supporting plates with different angles using (**a**) MCI-based semiautomatic segmentation method and (**b**) manual segmentation method. One-way repeated measures ANOVA showed no statistically significant difference in volume measurement of the same model at different placement angles (P > 0.05).

Different placement angles had no significant impact on the volumetric measurements with two segmentation methods (P > 0.05) (Fig. 1). Therefore, measured volumetric data of bone grafts in each model mounted on different supporting plates with two test methods were merged and means, minimum and maximum values of the volume measurements were shown in Table 2. The coefficients of variation representing the repeatability of the MCI-based segmentation method showed low values of 0.67% and 1.12% for S1, S2 model, and 0.65% for the R model. The corresponding values of the manual method showed high values of 0.95% and 3.65% for S1, S2, and 3.23% for R.

**Table 2:** Merged data of volume (mm^3^) measurements of bone grafts filled in vitro models positioned on different supporting plates.

	MCI-based			Manual		
	S2	S1	R	S2	S1	R
Mean	375.55	965.16	888.18	337.11	888.06	831.88
Standard deviation	4.21	6.42	5.79	12.32	8.48	26.86
Minimum	370.78	955.93	879.66	322.17	875.39	780.72
Maximum	381.67	976.19	891.99	354.17	898.18	870.96
Coefficient of variation (%)	1.12	0.67	0.65	3.65	0.95	3.23

As for the ex vivo models, the mean, minimum, and maximum values of the volume measurements obtained by the two test methods were shown in Table 3. The coefficients of variation representing the repeatability of the MCI-based segmentation method showed low values ranging from 0.93% to 1.21%. The corresponding values of the manual method showed high values ranging from 1.29 to 3.30%.

**Table 3:** Volume (mm^3^) measurements of bone grafts filled in ex vivo models using the test measurements.

	MCI-based				Manual			
	BD_5 mm−1.5_	BD_5 mm−3_	BD_6 mm−1.5_	BD_6 mm−3_	BD_5 mm−1.5_	BD_5 mm−_	BD_6 mm−1.5_	BD_6 mm−3_
Mean	98.20	97.68	139.98	140.09	94.08	93.22	136.96	137.63
Standard deviation	1.10	0.91	1.52	1.70	2.47	3.08	1.76	2.08
Minimum	96.78	96.18	138.16	138.61	90.76	88.89	134.90	135.27
Maximum	99.79	98.64	141.53	142.65	96.48	97.24	139.45	140.84
Coefficient of variation (%)	1.12	0.93	1.09	1.21	2.63	3.30	1.29	1.51

### Accuracy

The accuracy of the measured volumes using the MCI-based method compared with the control values was high and well below clinical significance (Tables 4, 5). For in vitro models, the highest systematic error of the manual and semiautomatic methods reached − 96.56 mm^3^ and − 40.26 mm^3^ for R, respectively. The relative systematic error ranged from − 7.23% (S2) to 1.09% (S1) for the MCI-based method and from − 16.73% (S2) to − 6.99% (S1) for the manual method. For ex vivo models, the highest systematic error of the manual and MCI-based methods reached − 4.91 mm^3^ (BD_5 mm−3_) and − 1.32 mm^3^ (BD_6 mm−1.5_), respectively. The relative systematic error ranged from 0.07% (${\text{BD}}_{{5\;{\text{mm}} - 1.5}}$) to − 0.93% (BD_6 mm−1.5_) for the MCI-based method and from − 2.60% (BD_6 mm−3_) to − 5.00% (BD_5 mm−3_) for the manual method.

**Table 4:** Differences in volume measurements (mm^3^) of the model R and S1–2 using the test and the control measurements.

	MCI-based			Manual		
	S2	S1	R	S2	S1	R
Mean of control method	404.82	954.77	928.44	404.82	954.77	928.44
Mean of test method	375.55	965.16	888.18	337.11	888.06	831.88
Systematic error	− 29.27	10.39	− 40.26	− 67.71	− 66.71	− 96.56
Relative systematic error (%)	− 7.23	1.09	− 4.34	− 16.73	− 6.99	− 10.40

The systematic error of the test method is given in cubic millimeters and percent.

**Table 5:** Differences in volume measurements (mm^3^) of ex vivo models using the test and the control measurement.

	MCI-based				Manual			
	BD_5 mm−1.5_	BD_5 mm−3_	BD_6 mm−1.5_	BD_6 mm−3_	BD_5 mm−1.5_	BD_5 mm−3_	BD_6 mm−1.5_	BD_6 mm−3_
Mean of control method	98.13	98.13	141.30	141.30	98.13	98.13	141.30	141.30
Mean of test method	98.20	97.68	139.98	140.09	94.08	93.22	136.96	137.63
Systematic error	0.07	− 0.45	− 1.32	− 1.21	− 4.05	− 4.91	− 4.34	− 3.67
Relative systematic error (%)	0.07	− 0.46	− 0.93	− 0.86	− 4.13	− 5.00	− 3.07	− 2.60

The systematic error of the test method is given in cubic millimeters and percent.

### Morphological evaluation

**Figure 2:**
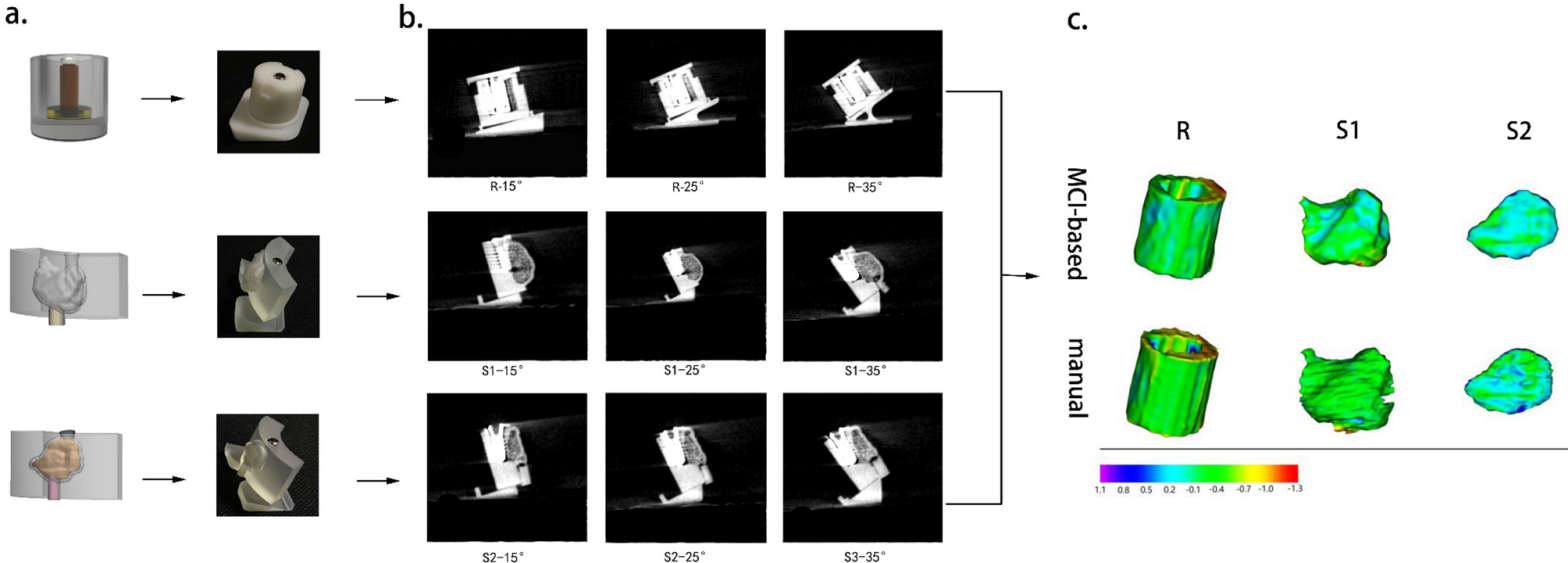
(**a**) Three 3D printed models exhibiting concentric cylinder geometry (R) and complex geometrical forms (S1/S2) imitating implant placement with simultaneous GBR procedure. (**b**) CBCT images of three models placed on three different auxiliary appliances. (**c**) 3D color map showing morphological deviation between the reference model and the 3D surface renderings using manual and MCI-based segmentation.

**Figure 3:**
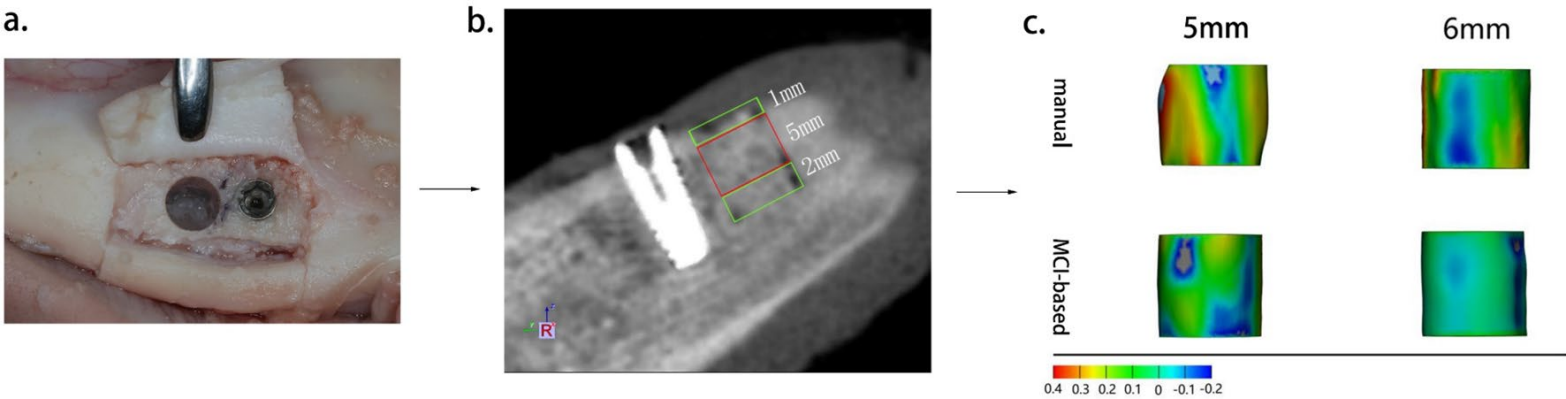
(**a**) Occlusal view of a cylindrical peri-implant bone defect. (**b**) CBCT images with the volumetric measurements of the 5 mm height of bone grafts in the augmented regions. (**c**) 3D color map showing morphological deviation between the 5 mm height of the ideal cylinder and the 3D surface renderings using manual and MCI-based segmentation.

Figures 2c and 3c showed the morphological differences between the 3D surface renderings of bone grafts created by the two segmentation methods and corresponding reference models. The results showed that the 3D surface renderings of bone grafts obtained by the MCI-based segmentation method had a lower deviation from the reference.

### Execution time

**Figure 4:**
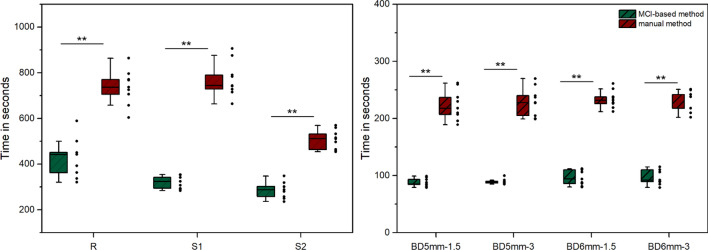
Box-and-scatter plot of the time it took to measure the volume of bone grafts in models with manual and MCI-based segmentation. Each data point represents one measurement. Manual segmentation took a significantly longer time than MCI-based segmentation.

Figure 4 showed the time consumed by the two segmentation methods for the measurements of bone grafts volume. The results indicated that the time for measurement with the MCI-based segmentation method was significantly less than that of the manual segmentation method (P < 0.001).

## Discussion

The present study aimed to test the MCI-based semiautomatic method with a manual method for the volumetric measurements of bone grafts around the implant. The computer-assistant calculation for the volume of space inside models for filling bone grafts was set as a reference in this study. The results demonstrated the investigated MCI-based method was more accurate than the manual method for volumetric measurement of bone grafts around the implant as shown by the smaller differences between the measured and actual volume for all models. Additionally, excellent repeatability of the MCI-based method was observed as shown by the lower coefficients of variation compared with the manual method for all models.

The small differences between measured and actual volume in all models showed the MCI-based method (lower relative systematic error for every model) was more accurate than the manual method. The reason for improving the accuracy of volumetric measurement was that the MCI algorithm could avoid the delineation of grafted bone on every slice and error accumulation of manual segmentation in slices with serious artifacts interference.

It was worth mentioning that a higher relative systematic error of volume measurement was found in the S2 model compared with R and S1 (S2: − 7.23%/− 16.73%, S1: 1.09%/− 6.99%, R: − 4.34%/− 10.40% for MCI-based and manual method, respectively ) using two methods. Owing to the concave feature of S2, the extent to which artifacts interfered with the outer boundary of bone grafts in S2 may be larger than that of the other two models. For ex vivo models, the regular cylinder model was chosen in this study because it was difficult to create complex geometric structures in ex vivo for measurements. Lower relative systematic errors were found for volumetric measurements of ex vivo models than in vitro models. Easy segmentation of cylinder form may be the reason.

The coefficients of variation for measurements in all models showed better repeatability of MCI-based (< 1.2%) than that of a manual method (0.95–3.65%). The results indicated the superior applicability of the MCI-based segmentation method and this was by the findings in our previous study^18^. The stability of the MCI algorithm may account for the improved repeatability of measurements.

As for the morphological evaluation of reconstructed 3D surface renderings and time management, 3D surface renderings of bone grafts obtained by the MCI-based method showed lower morphological deviation from reference models. This result suggested that the morphology of interpolated sequences generated by iteration MCI algorithm was indeed similar to the input slices without obvious oversize or undersize slices. Due to the reduction of the manual segmentation process, the MCI-based method took less time to measure compared with the manual method.

To the best of our knowledge, the semiautomatic method proposed in this study for volumetric measurements of bone grafts around the implant is quite different from semiautomatic segmentation methods mentioned in previous studies. Many semiautomatic segmentation methods have been proposed in the literature. These methods essentially concentrate on the region of interest segmentation on every slice and few are superior to manual segmentation^19^ in CBCT images without artifacts interference. It is reasonable to assume that the manual segmentation method will be more accurate than other semiautomatic methods in CBCT images affected by artifacts.

It should be noted that the MCI algorithm does not attempt to improve the quality of CBCT images, and the operation of our semiautomatic method is completely based on the existing CBCT. Many image-modifying algorithms like metal artifacts reduction algorithms and methods for altering the image capture process have been developed to reduce artifacts involved in CBCT imaging^8^. However, artifacts that were inherent in the CBCT images cannot be thoroughly eliminated and the effect of these methods was controversial.

According to the results, the clinical application of the MCI-based segmentation method is very promising. It could provide the possibility to assess the influence factors of GBR outcome by evaluating 3D alteration of bone grafts at different follow-up time points. As a non-invasive method, future investigations could examine the correlation between initial grafting contour and GBR radiological outcomes and further provide an explicit standard on how much contouring is needed for GBR procedures.

Some limitations should be identified in the present study. Firstly, the conclusion was based on results using in vitro setup. Blood clot formation could not be reproduced in such an in vitro setup. Besides, the accuracy of the MCI-based method depends strongly on how well the region of interest is shown in the CBCT images and it is not suitable in cases of strongly distorted images. If CBCT images are distorted severely, a slice that is less affected by the metal artifact within 4–5 slices cannot be found and the accuracy will be affected.

## Methods

### In vitro model

Three customized models (1 standard model and 2 models derived from clinical cases) for assessing bone grafts volume were fabricated for imitating surgical implant placement with simultaneous GBR procedure.

One model (R) was manufactured with simple concentric cylinder geometry (Fig. 2a). Within the cylinder, two hollow cylinders were designed to contain dental implant and bone grafts, respectively. A cover with a looping structure was manufactured to provide a protective layer for the entrance and control the volume of bone grafts in the cylinder. The clinical situation was simulated by placing an implant into the inner cylinder and filling the intact outer cylinder with bone grafts under the cover. The size of the outer cylinder for filling with bone grafts was 928.44 mm^3^ (rounded to two digits after comma).

Two models (S1/S2) exhibiting complex geometrical forms were created to mimic the clinical situation of implant placement with a simultaneous GBR procedure (Fig. 2a). These models were mathematically designed based on CBCT data from two patients receiving GBR treatment in the maxillary anterior region. The form with a curved structure was designed to imitate alveolar bone and a hollow cylinder in it was utilized to contain an implant. The volume of irregular structure for bone grafts filling was as 954.77 and 404.82 mm^3^ (S1/S2).

All implants placed in R, S1, and S2 were Straumann Standard Plus SLA implants with 4.1 mm diameter and 10 mm length (Institut Straumann AG, Waldenburg, Switzerland). Local bone augmentation space was filled with deproteinized bovine bone particles (Bio-Oss, 0.25–0.5 mg, Geistlich Biomaterials, Wolhusen, Switzerland).

For R, S1, and S2, auxiliary supporting plates were fabricated to simulate the spatial position of the dental implant when taking CBCT in clinical practice. Three different angles of bevels on the auxiliary supporting plates range from 15° to 35° (A1–3, B1–3). For CBCT scans, R1 was positioned on A1–3, while S1 and S2 were positioned on B1–3 (Fig. 2b). Therefore, each model was scanned three times.

### Ex vivo model

Two fresh porcine mandibles were sectioned by using a handsaw in a vertical direction to produce four bone sections. This was done to reduce the model size to facilitate implant insertion and GBR procedure. A crestal incision in the lower border of mandible was made and a full thickness mucoperiosteal flap was raised. The implant sites were prepared according to manufacturing instructions. The implants used were bone level implants (Alpha-Bio Tec, SPI, 3.3 × 10 mm, Petach Tikwa, Israel). Two types of implants were used in vitro and ex vivo models with an attempt to mimic the clinical situation of different types of implant placement with simultaneous GBR. As for the GBR procedure, two trephines with an internal diameter of 4.0/5.0 mm and an external diameter of 5.0/6.0 mm were used to initiate a cut to a depth of 8 mm as measured from the bone crest to create a cylinder of bone 1.5 or 3 mm mesially away from the implant site. The entire core was gently loosened and removed (Fig. 3a). Thus, four peri-implant cylinder-shaped bone defects were created: BD_5 mm−1.5_, BD_5 mm−3_, BD_6 mm−1.5_, BD_6 mm−3_. Each bone defect was augmented with demineralized bovine bone mineral (Bio-Oss, 0.25–0.5 mg, Geistlich Biomaterials, Wolhusen, Switzerland) and covered with collagen membrane (Bio-Gide, Geistlich AG, Wolhusen, Switzerland). Then mucoperiosteal flaps were repositioned and sutured.

The volume of interest was set as the middle part of the bone graft (5 mm). The 1 mm in the top and 2 mm in the bottom were excluded to avoid the unsmooth interface (Fig. 3b).

### CBCT scanning

All scans were performed using the CBCT machine (i-CAT Cone Beam Computed Tomography machine, USA). The datasets were obtained with a voxel resolution of 0.25 mm, a field of view (16 cm diameter/13 cm height), a tube voltage of 120 kV and a tube current of 5 mA. The data sets were exported in digital imaging and communications in medicine (DICOM) format.

### CBCT image-based volumetric measurement of bone grafts

In this study, manual and MCI-based segmentation methods were used to perform volumetric measurements and 3D reconstruction of bone grafts in models. The acquired CBCT DICOM datasets were imported into Medraw software (Image Medraw Technology Co., Ltd, Shanghai, China) for segmentation, iteration of MCI algorithm, 3D surface renderings reconstruction, and subsequent volume analysis of bone grafts. All measurements were performed by two doctors who had learned how to operate the software, and the mean value was recorded. Five repeated measurements of each CBCT image were performed using two different segmentation methods, respectively.

In the conventional manual segmentation method, bone grafts could be demarcated from models by density and structure. Each slice was displayed on computer monitor and drawing function was used to trace manually the perimeters of the bone grafts area on each coronal or sagittal section.

**Figure 5:**
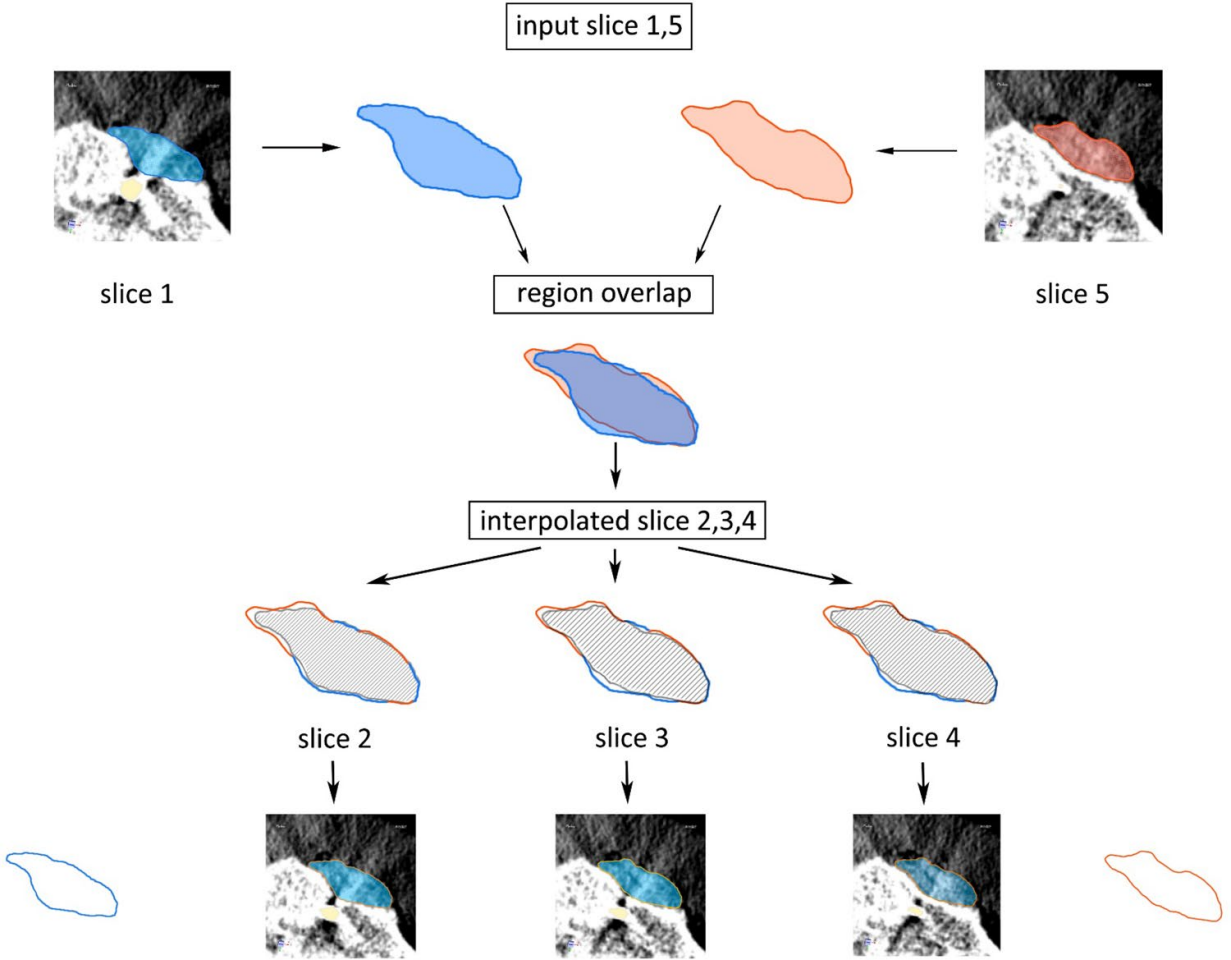
One iteration in the proposed morphological contour interpolation.

In the semiautomatic segmentation method, it mainly involved two steps as described in our previous study^18^. Briefly, perimeters of the grafted bone area were manually traced on coronal or sagittal slices without or with few artifacts’ interferences. Manually segmented slices were selected as the input slice of the MCI algorithm. Afterward, the iteration of the MCI process automatically computed a transition sequence between a pair of corresponding input slices which could gradually transform the shapes and elements equally similar to the input slices were selected. The schematic diagram of the MCI algorithm was shown in Fig. 5. After the segmentation procedure, the 3D surface rendering of bone grafts could be visualized in a separate 3D visualization window. The measurements of grafted bone volume were calculated by the built-in analysis module from segmented data.

## Outcomes

### Volumetric measurement

#### Repeatability

The repeatability of the test method was assessed by calculating the coefficients of variation of the repeated measurements for each model.

#### Accuracy

The actual volumes of bone grafts filled in vitro models (R, S1/S2) were assessed by combining the computer-aided calculation and the precision of the Lite600 3D printer (0.01 mm in linear dimension) (UnionTech stereolithography).

The actual volumes of bone grafts filled in ex vivo models were considered as the volume of a cylinder with 5 or 6 mm in diameter and 5 mm in height.

The computer-calculated volume values of bone grafts in vitro and ex vivo models served as a control, whereas the manual and semiautomatic segmentation methods represented the test measurements.

### Morphological evaluation of 3D surface rendering

Point-based registration was used to evaluate the accuracy of 3D surface renderings created by the two segmentation methods. Medraw software was used to measure the 3D deviation of the registered models for each reference point group on standard tessellation language (STL) files obtained from the CBCT scans. STL files of the computer-generated corresponding models were regarded as the reference and the 3D surface renderings obtained from two segmentation methods again served as the test.

### Execution time evaluation

The time needed to determine measurements for the two test methods was recorded and the comparison was performed.

## Statistical analysis

All analyses were performed with a standard statistical software package (SPSS version 21, Chicago, IL). Mean values and standard deviations were calculated.

The repeatability of the test method was evaluated by calculating the coefficients of variation of the repeated measurements for each model.

The accuracy of the test method was calculated by comparing the mean values of the test with the actual values. The difference between these values was presented as the systematic error of the test method. The relative systematic error of the test measurements was calculated by dividing the systematic error of the test measurements by the control values multiplied by 100.

One-way repeated-measures ANOVA was used to analyze the mean measured volume of R, S1, and S3 mounted on different supporting plates, and paired student’s t-test was used to assess the execution time of two segmentation methods. The level of significance was set at 5%.
